# Smoking among healthcare professionals is a contradictory behavior with public health implications: A narrative review

**DOI:** 10.18332/tpc/236154

**Published:** 2026-11-11

**Authors:** Andreea Nicoleta Mălăescu, Florin Dumitru Mihălțan

**Affiliations:** 1 Department of Cardio-Thoracic Pathology Carol Davila University of Medicine and Pharmacy Bucharest Romania; 2 Department of Pneumology III National Institute of Pneumophthisiology Marius Nasta Bucharest Romania

**Keywords:** smoking, tobacco, healthcare workers, medical personnel, physicians

## Abstract

Smoking among healthcare professionals remains a major public health challenge, affecting not only their well-being but also the credibility of medical practice and the effectiveness of tobacco cessation counseling. This narrative review examined smoking prevalence, associated factors, and counseling practices among healthcare professionals. The research was conducted by accessing databases such as PubMed, Scopus and Web of Science, including articles published between 2012 and 2025. The analyzed samples ranged from institutional cohorts to national surveys, and included doctors, nurses, resident physicians, and other medical staff. Original studies that assessed the prevalence of smoking among medical staff, sociodemographic characteristics, attitudes and practices regarding counseling patients to quit smoking, were selected. Non-original articles that did not provide relevant background or contextual information, studies that did not clearly specify the professional status of respondents or those whose data were irrelevant to the subject or of doubtful clarity, were excluded. A total of 68 studies were selected, including 60 original studies. The findings underscore the vast heterogeneity in terms of study design, geographical distribution, and population characteristics. The prevalence of smoking varies significantly depending on professional category and geographical area. Among physicians, reported prevalence ranged from approximately 0.5% in Malaysia to over 50% in Turkey. Smoking is more common in men, younger professionals and auxiliary medical professions. Although most health professionals are aware of the risks of tobacco smoking, the practice of counseling patients remains suboptimal, influenced by lack of time, specific training and their own smoking behavior. The results highlight a wide variability in smoking prevalence among medical staff, depending on the professional category, gender, age, and geographical context, as well as the existence of gaps in patient counseling practices for smoking cessation. The heterogeneity of the included studies and the different methodologies underscore the need for further research to provide solid evidence and clarify the determinants of smoking among medical personnel.

## Introduction

The study of the influence of chronic smoking on general health is still a priority interest worldwide, as smoking is an important risk factor for multiple diseases – particularly those affecting the respiratory, cardiovascular, and oncological systems^1-3^. Recent studies have shown important differences in smoking prevalence according to geographical area, gender, and socioeconomic context^4-7^. According to the World Health Organization (WHO), around 1.3 billion people worldwide currently use tobacco products, with more than 80% coming from low- and middle-income countries^8^. These differences are also important in healthcare professionals, given their role in promoting smoking prevention.

As medical staff represent a subgroup of the population that plays a crucial role in health promotion and disease prevention, the prevalence of tobacco smoking among them is of particular significance. Healthcare professionals, regardless of their specialty, serve as role models, not only for patients, but for the entire community, and their health-related behaviors influence the credibility of preventive messages^9^. In this context, in-depth knowledge of the health consequences of smoking is becoming essential to raise awareness among medical staff about the risks associated with this widespread addiction^10^. Therefore, it is important to mention the vast diversity of conditions associated with tobacco use, in order to understand the magnitude of the problem^3,11^. Smoking affects multiple organ systems, particularly the respiratory system^1,3,11^. It remains a major risk factor for lung cancer and chronic obstructive pulmonary disease (COPD)^1,12^ and is also associated with smoking-related interstitial lung diseases, including respiratory bronchiolitis-associated interstitial lung disease (RB-ILD), desquamative interstitial pneumonia, and pulmonary Langerhans cell histiocytosis^2^. In addition to respiratory pathology, smoking has been associated with cardiovascular disease, metabolic disorders, depression, and oral health problems^3,13-15^.

Moving to smoking among medical staff, it is important to emphasize both their role in treating disease and their position as role models in society. In public health, the behavior of healthcare professionals, particularly in relation to smoking, is of significant importance. The non-smoking status of medical staff is a priority, which increases their credibility in promoting smoking cessation and strengthening their role in promoting a healthy behavior. Evidence shows that non-smoking healthcare professionals are more likely to offer advice on quitting smoking, which may be more effective in patients' attempts to quit^9,16^.

Although information about the risks of smoking is widespread and there are strong anti-smoking policies, there remains a high prevalence of smokers among medical staff, despite the fact that their behavior directly influences public health efforts^9,16^. People who work in healthcare facilities and smoke compromise their ability to promote smoking cessation, undermine their influence in health promotion and send mixed messages to the community^16-18^.

Smoking prevalence among healthcare workers varies considerably according to geographical area and professional category^9,16,19^. In high-income countries the prevalence of smoking is usually lower among medical staff compared to the general population, while in low- and middle-income countries, it may be similar or even higher^9^. Increased smoking prevalence has been reported among nurses and auxiliary healthcare workers, particularly among males, younger professionals, and those exposed to workplace stress^9,16^.

Although there are extensive data on smoking in the general population, the literature devoted to tobacco use among medical personnel is relatively limited and often inconsistent. In addition, data on the use of new nicotine-based products, such as e-cigarettes and heated tobacco devices, are fragmented among healthcare professionals. These limitations highlight the need for an up-to-date analysis to summarize existing data and clarify current patterns of tobacco use among healthcare professionals. Therefore, this narrative review aimed to summarize the available evidence regarding smoking among healthcare professionals. The review examined prevalence of smoking across different professional categories, from doctors and nurses to medical faculty and pharmacists, and explored factors associated with smoking behavior, including gender, age, education level, and workplace-related influences, such as professional stress, daily routines, and social influences. It also reviewed barriers related to smoking cessation counseling among healthcare professionals.

For the selection of relevant sources for this review, a search was conducted in internationally recognized databases, which included PubMed, Scopus and Web of Science, until May 2025. Studies published in English and French were considered. The search strategy included keyword associations such as ‘smoking’, ‘tobacco use’, ‘prevalence’, ‘nicotine’, ‘healthcare personnel’, ‘smoking cessation interventions’. An example of expression for logical queries was (‘smoking’ OR ‘nicotine’ OR ‘cigarette’ OR ‘tobacco’) AND (‘healthcare personnel’ OR ‘medical staff’ OR ‘nurses’ OR ‘physicians’) AND ‘prevalence’. The search was filtered to include studies published between 2012 and 2025 to ensure current data and approaches to smoking among healthcare professionals. However, one earlier study of particular relevance to smoking behaviors among medical students was also included.

The inclusion criteria mainly focused on cross-sectional original studies with a clearly described methodology that analyzed the prevalence of smoking among medical staff, investigated behaviors and attitudes of healthcare professionals towards tobacco, or assessed the effectiveness of anti-smoking intervention and counseling programs applied in health institutions. The selected studies addressed both quantitative data (e.g. prevalence, amount of tobacco consumption) and qualitative data (e.g. perceptions, barriers, motivations).

The exclusion criteria were represented by studies that presented unclear or irrelevant data related to the subject, non-original articles that did not provide relevant background or contextual information (e.g. editorials, letters to the editor), studies without full-text availability, and those that do not clearly mention the professional status of respondents.

The selected studies were screened and analyzed by a single researcher. Duplicates were identified and removed manually. No automated screening software was used. The titles and abstracts were initially revised for eligibility analysis, followed by evaluation of the full texts. The data were extracted manually, and the results were later compared according to the professional category, sociodemographic factors, geographical context and barriers to smoking cessation.

## Developments

### Study characteristics

A total of 68 studies were selected, including 60 original studies. Among the original studies, cross-sectional studies represented the predominant study design (n=42), followed by observational pre-post intervention studies (n=7). In addition, the review included four qualitative studies, three randomized controlled trials, two retrospective observational studies, one prospective observational intervention study, and one population-based microsimulation study. Most studies were conducted in Europe (n=27) and Asia (n=18), whereas fewer originated from North America (n=6), Oceania (n=4), Africa (n=3), and South America (n=2). Studies examined smoking prevalence (Tables 1 and 2), associated sociodemographic factors (Table 3), and practices regarding smoking cessation counseling among doctors, medical students, nurses, pharmacists, and other healthcare staff^17-84^.

**Table 1:** Prevalence of smoking among doctors and medical students by geographical region and publication year (studies published between 2015 and 2025)

Authors Year Country	Data collection period	Population	Sample size	Study design	Smokers (%)	Non-smokers at the time of study	
						Ex-smokers (%)	Non-smokers (%)
Zinonos et al.^83^ 2016 Cyprus	2008	Doctors	119 doctors	Cross-sectional	28.6	16	55.5
Edwards et al.^41^ 2018 New Zealand	2013	Doctors	7065 male doctors 5619 female doctors	National census – Administrative data analysis	2	~13	>85
Pianori et al.^53^ 2017 Italy	2006, 2011, 2015	Doctors	475 healthcare professionals	Cross-sectional	27.27	72.73	
Sonmez et al.^29^ 2015 Turkey	-	Doctors	1182 PCPHs	Cross-sectional	34.4	14	51.5
Lina et al.^38^ 2016 Italy	-	Doctors	285 physicians	Cross-sectional	14.1	23.5	62.4
Minardi et al.^20^ 2021 Italy	2014–2018	Doctors	169678 working-age respondents1253 medical doctors	Cross-sectional, National surveillance – PASSI	16	84	
Yen et al.^21^ 2022 Vietnam	2017	Doctors	3343 healthcare workers	Cross-sectional	13.3	86.7	
Wang et al.^42^ 2021 China	2018	Doctors	1050 doctors 1046 valid questionnaires	Cross-sectional	14.7	85.3	
Zorlu^37^ 2022 Turkey	2019	Doctors	21 specialist physicians (121 participants overall)	Cross-sectional	52.4	9.5	38.1
Ling et al.^36^ 2022 Malaysia	2020	Doctors	383 participants	Cross-sectional	0.5	2.6	96.9
Priano et al.^33^ 2024 Italy	2023	Doctors	1561 healthcare workers	Cross-sectional, Occupational records	17.8	82.2	
Hariri et al.^50^ 2025 Syria	2023–2024	Resident physicians	294 resident doctors	Cross-sectional	22.1	77.9	
Pianori et al.^53^ 2017 Italy	2006, 2011, 2015	Medical and nursing students	22 students (475 participants overall)	Cross-sectional	45.45	54.55	
Shrestha et al.^78^ 2020 Nepal	2019–2020	Medical students	113 participants	Cross-sectional, Global Health Professional Students Survey	30.1	69.9	

PCPHs: Primary care physicians.

**Table 2:** Prevalence of smoking among nurses by geographical region and publication year (studies published between 2015 and 2024)

Authors Year Country	Data collection period	Sample size	Study design	Smokers (%)	Non-smokers at the time of study	
					Ex-smokers (%)	Non-smokers (%)
Zinonos et al.^83^ 2016 Cyprus	2008	392 nurses	Cross-sectional	28.1	5.9	66.1
Barbosa et al.^22^ 2015 Brazil	2008–2009	228 nurses and nursing technicians (1808 health workers overall)	Cross-sectional	12.8	87.2	
Schneider et al.^84^ 2019 Scotland	2008–2012	471 nurses	Cross-sectional, Scottish Health Survey	17.4	30.6	52
Edwards et al.^41^ 2018 New Zealand	2013	2988 male nurses 36138 female nurses	National census – Administrative data analysis	8	26.4	65.6
Sonmez et al.^29^ 2015 Turkey	-	1063 PCNs	Cross-sectional	30.7	10.1	59.3
Bialous et al.^68^ 2017 Czech Republic, Poland	2014	436 nurses	Cross-sectional	13.82	25.35	60.83
Pianori et al.^53^ 2017 Italy	2006, 2011, 2015	475 healthcare professionals	Cross-sectional	33.65	66.35	
LeBlanc et al.^55^ 2020 Canada	2014–2016	406 nurses	Observational	8	11.2	80.2
Malone et al.^52^ 2016 Australia	2015	26 nurses	Qualitative focus groups and survey	4	22	73
Rezk-Hanna et al.^58^ 2018 Central and Eastern Europe	2015–2016	81 nurses	Focus group	65	35	-
Petersen et al.^56^ 2020 Czech Republic, Hungary, Romania, Slovakia, Slovenia	2015–2016	82 nurses	Qualitative using focus groups	65.4	34.6	Not reported (sampling excluded never smokers)
Taniguchi et al.^70^ 2022 Japan	2019–2020	289 nurses	Longitudinal	5.6	26.4	68
Cousin et al.^26^ 2022 France	2020	654 nurses	Cross-sectional online survey (ALADDIN)	30.7		69.3
Priano et al.^33^ 2024 Italy	2023	1561 healthcare workers	Cross-sectional, Occupational records	29.7	70.3	

PCNs: Primary care nurses.

**Table 3:** Sociodemographic factors associated with smoking among healthcare personnel (studies published between 2013 and 2025)

Authors Year Country	Data collection period	Sample size	Sex	Age	Education level
Barbosa et al.^22^ 2015 Brazil	2008–2009	1808 health workers	Male sex was associated with a higher smoking prevalence (PR=1.75; 95% CI: 1.36–2.25)	Not reported	Smoking associated with lower level of education in auxiliary professions
Ferrante et al.^80^ 2013 Italy	2012	422 students	Smokers Males: 46.8% Females: 33.2%	<24 years: 43.1% smokers ≥25 years: 33.8% smokers	Higher smoking in health professional school students vs medical school students (49.2% vs 29.5%)
Edwards et al.^41^ 2018 New Zealand	2013	7065 male doctors 5619 female doctors 2988 male nurses 36138 female nurses	Current smokers Male doctors: 2% Female doctors: 2% Male nurses: 9% Female nurses: 8%	Current smokers Doctors: 15–24 years: 2.2% 25–44 years: 1.8% 45–64 years: 2.3% ≥65 years: 1.7% Nurses: 15–24 years: 6.6% 25–44 years: 7.9% 45–64 years: 8.3% ≥65 years: 6.6%	
Pianori et al.^53^ 2017 Italy	2006, 2011, 2015	475 healthcare professionals	Current smokers Male: 32.96% Female: 35.57%	Average age of smokers: 39.94 years Mean age at smoking onset: 18.1 years Mean age at smoking cessation: 30.8 years Smoking decreased with increasing age (OR=0.97; 95% CI: 0.95–0.99; p<0.01)	Higher smoking prevalence among students (45.45%) compared to medical doctors (27.27%)
LeBlanc et al.^55^ 2020 Canada	2014–2016	406 nurses	94.4% of sample were female 8% currently smoke (daily or occasionally)	Average age of smokers: 45.4 years Average age of non-smokers: 42.6 years	Smokers more likely to have college degree (71.9%)
Minardi et al.^20^ 2021 Italy	2014–2018	169678 working-age respondents 1253 medical doctors	Smokers Male doctors: 18.4% Female doctors: 13.9%	Smokers doctors: 18–34 years: 24.4% 35–49 years: 13.7% 50–65 years: 14.7%	Smokers Less than high school: 37.4% High school: 26.3% College graduates: 21.9%
Rezk-Hanna et al.^58^ 2018 Central and Eastern Europe	2015–2016	81 nurses	65% of participants were current smokers 94% of participants were female nurses	Average age of participants: 43 years	64.2% Diploma level nursing education 7.4% Bachelor’s degree 16% Master's degree
Lina et al.^38^ 2016 Italy	-	285 physicians	Smokers Male doctors: 15.7% Female doctors: 12.2%	Smokers: 20–50 years: 11.8% >50 years: 17.9%	Physicians in a comprehensive cancer center
Yen et. al.^21^ 2022 Vietnam	2017	3343 healthcare workers	Smokers Male healthcare workers: 22.2% Female healthcare workers: 0.5%	Among smokers: 16.3% were aged 18–29 years, 38.8% 30–39 years, 27.8% 40–49 years, 16.3% 50–59 years, and 0.8% ≥60 years	Higher smoking prevalence among doctors compared to other professions
Gücük et al.^23^ 2020 Turkey	2018	387 health workers	Smokers Male: 40.4% Female: 18.4%	Highest proportion: 30–39 years (45.2%)	Not reported
Shrestha et al.^78^ 2020 Nepal	2019–2020	113 participants	Current smokers Male: 23% Female: 7.1%	Among current smokers, 22.1% were aged 19–24 years and 7.1% were aged 25–29 years	Third-year medical students in tertiary institution
Cousin et al.^26^ 2022 France	2020	1238 participants	Daily smokers Male healthcare workers: 24.4% Female healthcare workers: 20.6%	Age 20–34 : 23.7% daily smokers	Not directly reported; the ‘other’ professional group - The highest daily smoking prevalence (40.2%)
Ruiz et al.^35^ 2023 Spain	2022	802 respondents	6.6% current smokers Women 66% Men 34%	Median age of smokers: 48 years Highest smoking: age 25–35 (10.6%)	High level (physicians from the Spanish Society of Pulmonology and Thoracic Surgery – SEPAR)
Priano et al.^33^ 2024 Italy	2023	1561 healthcare workers	Smokers Male: 28% Female: 23.4%	Average age of smokers: 37.6 years Average age of non-smokers: 36.8 years	Higher smoking prevalence among non-healthcare technicians
Hariri et al.^50^ 2025 Syria	2023–2024	294 resident doctors	Smokers Male doctors: 39.5% Female doctors: 10.3%	Smokers: 26.75 years Non-smokers: 26.33 years	High level (resident physicians)

OR: odds ratio. PR: prevalence ratio. CI: confidence interval.

### Smoking prevalence among healthcare professionals

A 2014–2018 study in Italy identified a 23% prevalence of smokers among healthcare workers, compared with a 28.6% prevalence in other categories of employees. The lowest smoking prevalence was identified among doctors (16%), while the prevalence among auxiliary medical staff was 25.3% (95% CI: 23.7–26.9)^20^. However, a study in Vietnam identified the highest prevalence among doctors (13.3%), followed by among technicians and administrators, illustrating the variability in medical staff habits likely to be determined by geographical influences, in a country with a smoking prevalence in the general male population at the time of 45.3%^21^.

Similarly, a study in Brazil found a slightly higher prevalence of smoking among public health workers (15.7%) compared to the general territorial population at the time (15.2%). Most smokers were identified among the staff with supervision duties (24.3%), and the lowest among nurses (12.8%) and physicians (7.9%)^22^.

In Morocco, Badri et al.^39^ reported that among medical and paramedical staff, 75.5% were non-smokers, about 8% were ex-smokers, and only about 16% were active smokers, with male predominance and an average consumption of 9 cigarettes per day.

In Turkey, Gucuk et al.^23^ identified that almost a quarter of healthcare workers were active smokers, and almost 76% of non-smokers were passively exposed to smoking at work. Moreover, a smoking history of over 10 years was associated with lower health responsibility, interpersonal relationships and a lower level of self-fulfillment.

#### Factors associated with smoking behavior

The increased level of stress at work is the most commonly mentioned factor underlying the increase in the prevalence of tobacco use. Long working hours, shift work, and emotional demands may prompt medical staff to use smoking as a coping mechanism for stress and anxiety^24,25^. During periods of prolonged stress, there is a tendency to increase smoking. An example is the significant impact of the COVID-19 pandemic on tobacco consumption. In the pandemic period, smoking was the most prevalent psychoactive substance-related habit, with about 8.6% of respondents working in health facilities in Paris reported a significant increase in smoking compared to the pre-pandemic period, bringing the prevalence of smoking among staff to 21.4%^26^. There is also evidence that depression, or poor emotional well-being, is a risk factor for starting and continuing to smoke^16^.

#### Smoking cessation counseling and barriers

A study conducted in Indonesia investigated the association between smoking status of medical staff and tobacco counseling by applying the 5As model (Ask, Advise, Assess, Assist, Arrange)^27^. The majority of participants were non-smokers (81.7%). It was found that although all elements of the 5As model were applied consistently, the smoking history question (‘Ask’) was implemented most often^27^. It is important to note that the statistical analyses did not reveal significant associations between the smoking status of medical staff and the degree of application of the 5As model, suggesting that their own behaviors do not directly influence the involvement of medical staff in anti-tobacco counseling^27^.

Among the barriers in advising patients on smoking cessation are the perception that smoking is not the primary reason for a medical consultation, the belief that there are no effective cessation methods, the idea that patients prefer to quit on their own^28^, the lack of time, and the absence of financial compensation for this activity^29^.

It is also worth mentioning the lack of adequate training of medical staff, identified globally. Although most medical faculties cover the health effects of smoking, practical training activities for smoking cessation counseling have significant gaps. Of the Faculties of Medicine in the United Kingdom (UK), only about one-third provide practical instruction for students, and, on tests, half do not include smoking subjects^30^.

These shortcomings lead to low self-efficacy of young doctors in relation to smoking cessation counseling^31^. Interactive training, standardized patient encounters, and role-playing exercises may be more effective and have a greater impact on the medical capacity for smoking cessation counseling^32^.

#### Knowledge and attitudes regarding e-cigarettes

Another problem identified includes knowledge of medical staff about e-cigarettes. The problem is all the more important as there are health workers who support smoking cessation by switching from conventional cigarettes to e-cigarettes^28^.

In a hospital in Northern Italy, the percentage of e-cigarette users among all smokers was almost a quarter (21%)^33^. Another issue to consider is the impact they have on the environment, with studies proving the association between passive exposure and respiratory disease manifested by dyspnea, wheezing, bronchitis, and asthma exacerbations^34^. Among respondents, 76.6% consider electronic cigarettes unsafe and ineffective in smoking cessation. It has also been reported that young professionals are less informed about new products, and 29% of health workers are unaware of these devices^35^.

### Smoking among doctors

#### Smoking prevalence and associated factors

Smoking prevalence among doctors varies considerably across countries (Figure 1), ranging from approximately 0.5% in Malaysia^36^ to more than 50% in Turkey^37^. However, several studies suggest a downward trend in smoking among physicians. For example, a study conducted in Italy using data from the PASSI surveillance system between 2014 and 2018 identified a reduction in daily smoking among doctors of more than 40%, from 20.8% in 2014 to 11.5% in 2018. This decrease was almost twice as large as that observed in the general population, although the difference was not statistically significant due to the small sample size^20^.

**Figure 1:**
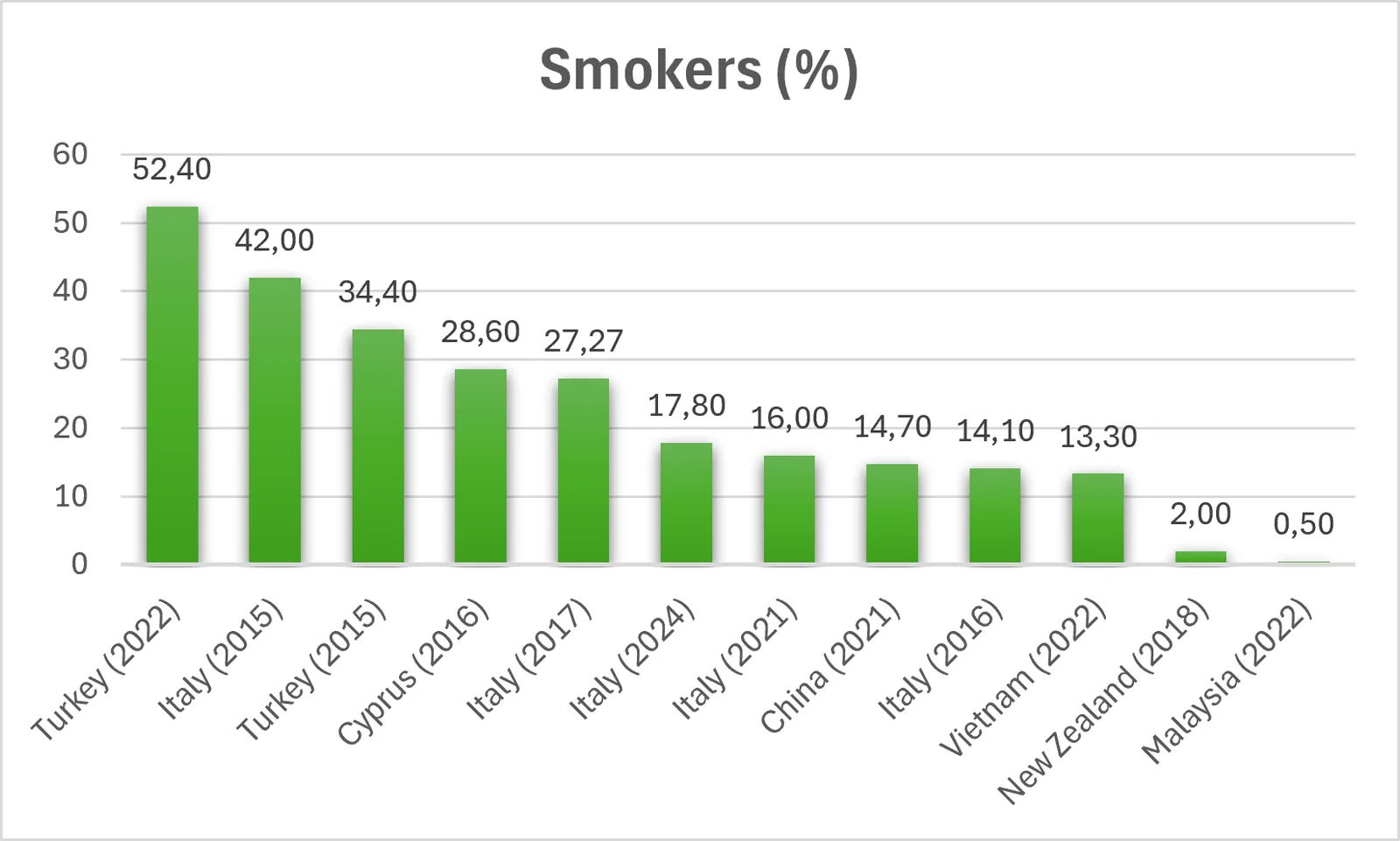
Geographical variability in smoking prevalence among doctors based on cross-sectional studies and national surveys published between 2015 and 2024 across multiple world regions. Percentages represent current smokers at the time of data collection

Lina et al.^38^ analyzed the smoking habits of doctors from an Italian oncology center, as well as their clinical practice related to counseling patients for smoking cessation and professional training in this regard. Among respondents, only 14% were active smokers, 62% never smoked and 24% were former smokers. Although nicotine addiction was low, with most smoking a maximum of 10 cigarettes per day, with a third of smokers declaring the desire to quit, a concerning pattern was the continued incidence of smoking within hospital facilities^38^.

However, there are areas where the prevalence of smoking among medical staff is extremely high, with a study conducted on employees of a hospital in Central Italy identifying a smoking prevalence of around 42% among doctors and approximately 43% among nurses, which is higher than in the general population^40^. Of those, 30% said they also smoked at work, while three-quarters said they wanted to quit smoking, but conflicting behaviors persist. The risky smoking behavior among medical staff can compromise their role as smoking cessation counselors^40^.

Another study in Turkey that included family doctors found that while 96.2% thought they were models for patients, 34.4% were smokers. Only 33.8% regularly ask patients whether they smoke, and only 13.1% advise the patient to quit^29^. Among respiratory professionals, the prevalence is lower, with ~5% of pneumologists in Spain smoking^35^.

Among physicians, smoking was more prevalent among males and younger professionals. The downward smoking trend of aging doctors, which automatically includes the acquisition of superior medical experience, probably arose due to awareness of the risks to which they are exposed^20^. Auxiliary medical staff had a higher trend of nicotine dependence, associated with economic difficulties and lower level of education, with greater vulnerability observed among socioeconomically disadvantaged groups^20^.

Common factors contributing to tobacco use among healthcare workers include break periods, workplace meetings^39^, concurrent alcohol consumption, frequent coffee intake^33^, and alternating day shifts–night shifts^26^.

A positive example to follow is that of New Zealand, which has a goal called ‘Smoke-free 2025’, defined by reducing the prevalence of smoking below 5% by 2025, which was achieved among doctors, while almost all categories of nurses were on the right track^41^. A study based on the 2013 New Zealand census showed a significant decrease in smoking among health professionals. In 2013, only 2% of doctors and 8% of nurses were active smokers, compared to 2006, when 4% of doctors and 13% of nurses smoked^41^. However, the situation is not as good among the medical staff in psychiatric wards, where 18% of nurses smoked. This unequal distribution underlines the need to implement targeted interventions for smoking recovery in this group^41^.

#### Smoking cessation counseling practices

Worryingly, most attempts at smoking cessation have been made without professional psychological or pharmacological support, highlighting the tendency to underestimate the complexity of nicotine addiction even in healthcare professionals^38^. Most doctors give up by personal will (about 67%), adopting a routine of physical activity or by nicotine substitutes^39^. Another problem was identified by a study conducted in a province of China, where only half of doctors knew the existence of smoking cessation drugs, and only about 5% prescribed drugs for this purpose^42^.

#### Interventions and smoking cessation programs

A recent example of effective intervention for the purpose of quitting smoking among medical staff is the Stop Smoking Promotion (SSP) program, which included an intensive 6 h course for employees willing to quit smoking, held during working hours in an Italian hospital and aimed at restructuring beliefs related to nicotine addiction^43^. After 2 years, 37.5% of participants had quit smoking. Moreover, within 1 year of the intervention, it has been reported to reduce medical absenteeism by 85%, results supporting the feasibility and cost-effectiveness of interventions for the smoking cessation of medical personnel^43^.

The ‘STOP SMOKING’ program, which has been running in Romania since 2007, consists of counseling services offered by pneumologists and psychologists specialized in smoking cessation^44^. It should be emphasized that although most doctors know the association of smoking with lung cancer (98.2%) and coronary heart disease (92.8%), the level of knowledge of other negative effects, such as osteoporosis (56.2%) or erectile dysfunction (60.9%), is significantly lower^42^. One aspect worth mentioning is the behavior of family doctors; a Lithuanian study identifying that only 23% always ask patients about smoking, 15% prescribe nicotinic replacement, and only 6% prescribe varenicline or bupropion^45^. In Malaysia, it was identified that family physicians had poor knowledge of smoking counseling and smoking cessation methods (62.4%), although almost 90% had attended smoking cessation courses^36^. The most common gaps were in the inefficient discussion of barriers to quitting smoking, the lack of an effective follow-up plan and the ineffectiveness of the patient-centered approach^46^.

On the other hand, the desire for training in smoking cessation counseling is a positive element. A study conducted among employees of an Oncology Centre in Italy found that, although only 6% had already attended specialized courses, nearly half of the physicians expressed a desire to be trained. A gratifying aspect is that their own smoking behavior did not significantly impact clinical practice^38^.

Around 86% of doctors ask patients about smoking habits, but only 50% offer advice on smoking. Obstacles identified in smoking cessation counseling of patients include the lack of time, fear of adding additional stress to patients already burdened by their primary disease, lack of skills, and, in the case of oncological patients, lack of conviction that they can be persuaded to quit smoking^38^.

A study conducted in a province of China found that counseling practices are more frequent among pediatricians, while surgeons are less involved in such interventions, a finding associated with a higher prevalence of smoking in this group^42^.

The problem of anti-smoking counseling is all the more important as it occurs in clinics specialized in lung diseases. The study conducted at Royal Hobart Hospital highlights a deficiency in initiating smoking cessation therapies among patients hospitalized for acute COPD exacerbations, with only about 35% of patients receiving some form of treatment for smoking cessation^47^. Combination nicotine therapy was given in only 6.6% of patients, and only 1.6% were treated with varenicline^47^.

A study in Russia even showed that pneumologists did not record the smoking status in electronic medical records at all, along with anesthesiologists, physical medicine and otorhinolaryngology. The highest frequency of records, about 50%, was observed in cardiologists, gynecologists and hematologists^48^. In Spain, however, 95% of pneumologists ask patients about tobacco use^35^.

Multiple factors are associated with the lack of consistent smoking cessation practices among physicians. These include inadequate training, limited access to educational resources (such as patient leaflets), absence of nicotine replacement options in some clinics^36^, and a general lack of confidence in prescribing cessation pharmacotherapy^47^. Additional barriers include staff overwork, concerns about damaging the patient–provider relationship, reluctance to refer patients to psychological support, and the prioritization of other health problems^49^.

#### Smoking among resident physicians

Resident physicians represent an important part of healthcare professionals and often have prolonged and close contact with patients. A study conducted at a university hospital in Syria evaluated smoking prevalence and patterns among resident physicians and found a smoking prevalence of around 22.1%^50^. Although the rate is lower than in the Syrian general population (about 38%), it remains significant and higher than in some European countries. The percentage was significantly higher among men (about 34%) compared to that among women (about 10%). The highest prevalence was found in surgical specialties (33.9%) and oncology (75%), and among residents in later years of training^50^. Shortness of breath, productive morning cough, depression, and insomnia were identified symptoms in up to a quarter of smokers with lower frequency and in passive smokers^50^. Other, less common symptoms included tremor, abdominal cramps, palpitations, weight loss, and sweating, which may impair professional performance. However, increased stress, increased clinical activity time and low personal satisfaction have been associated with persistence of the smoking habit^50^.

Another study conducted in an Australian rural psychiatric institution looked at the impact of an educational program on smoking cessation counseling on the training of resident doctors^51^. Its environment has been an ideal framework of practical experience given that smoking has a high frequency in psychiatric patients. Subsequent to the program, it was noted that resident physicians developed considerably their attitudes towards nicotine addiction, prescribing treatments for smoking cessation and behavioral technique^51^.

### Smoking among nurses

#### Smoking prevalence and sociodemographic characteristics

The prevalence of smoking among nurses varies significantly between countries (Figure 2), ranging from approximately 4% in Australia^52^ to over 43% in Central Italy^40^, with some countries exceeding the prevalence of smoking in the general population^54^.

**Figure 2:**
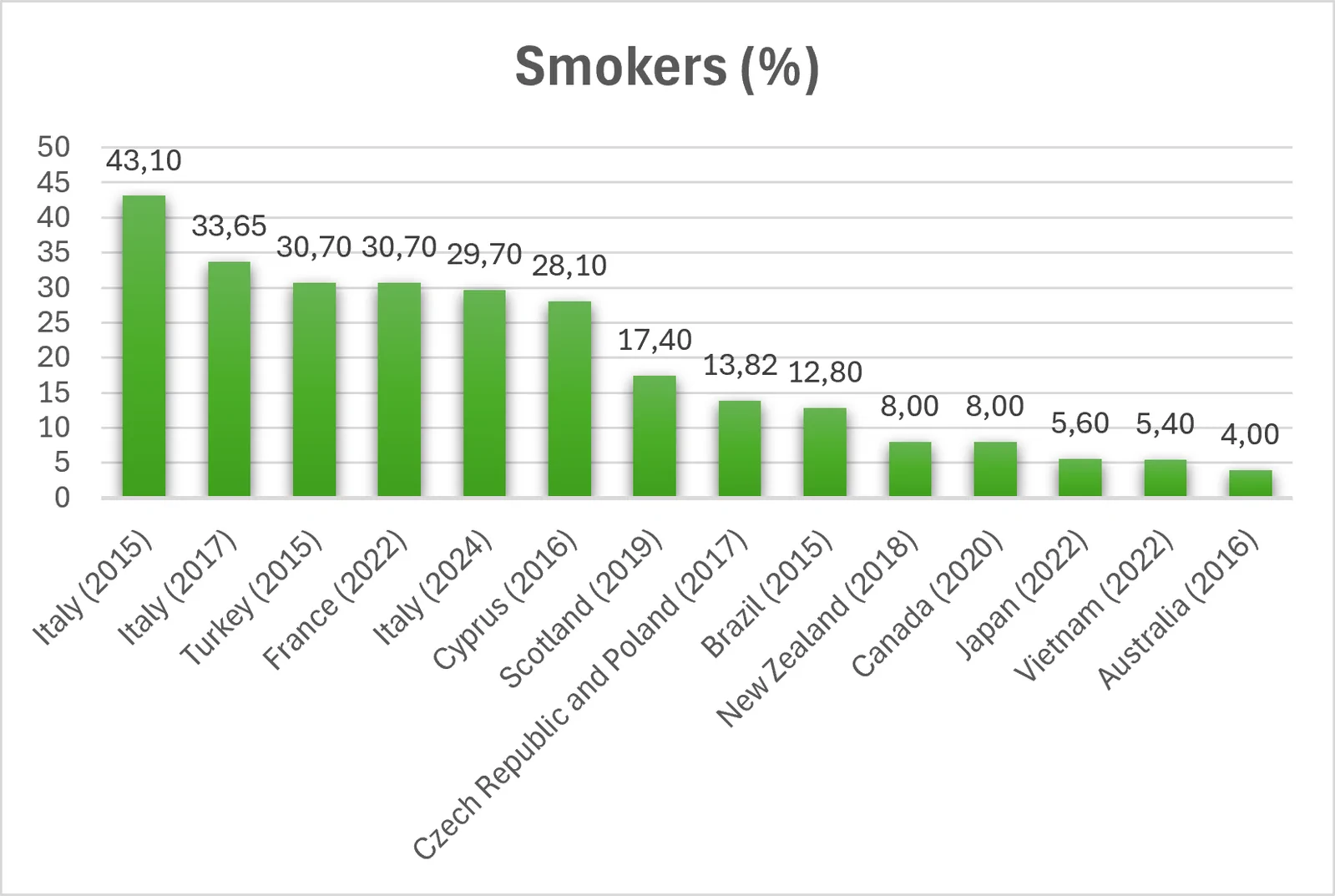
Geographical variability in smoking prevalence among nurses, based on cross-sectional studies and surveys published between 2015 and 2024 across multiple world regions. Percentages represent current smokers at the time of data collection

LeBlanc et al.^55^ identified that the prevalence among nurses in Ontario, Canada, was 8%, under the national prevalence of 13% in Canada, being higher in rural hospitals, 34.4% of smokers coming from rural areas, compared to 18.7% of non-smokers. In this study, marital status was the only factor significantly associated with smoking, as married women were less likely to be smokers.

#### Psychological and social factors associated with smoking

Nurses who smoke fear losing credibility in front of patients, experiencing feelings of guilt, shame and frustration, leading to the tendency to hide their smoking habit from patients (by using perfume or brushing their teeth to mask the odor) and minimizing the risks of smoking, which may either discourage patient counseling or lead to a reduction in the number of cigarettes smoked in a day or even the definitive smoking cessation^56,57^. There was also a tendency to share smoking-related experiences after quitting, with patients illustrating a model worth following^56^.

The main barriers identified for quitting smoking among nurses have included the association between professional stress and nicotine addiction, the presence of other smokers or environmental stimuli at work or at home (coffee breaks, post-meal periods, morning routines, cigarette smell), erroneous perceptions about the negative effects of smoking, post-partum relapse^58^. Up to 90% of women who quit smoking during pregnancy resume smoking within the first year after childbirth, endangering their and children’s health^59^. We also mention smoking as a method of facilitating social interaction and the sense of belonging in a group^56^.

Another problem they face in the smoking cessation includes the tendency of colleagues to undermine efforts by irony or by inviting smoking, predominantly smoking cessation attempts being solitary experiences without significant support^56^. Additionally, there is the perception that the medical staff who quit smoking remain without this stress management support and with additional responsibilities towards smokers, with non-smokers having fewer breaks at work^56^. A study of 82 nurses in Central and Eastern Europe identified smoking as a way of coping with stress, and associated smoking with work breaks, resulting in conflicts between smokers and non-smokers^56^.

The prevalence of smoking remained high even among nurses with a high level of medical knowledge about the risks they are exposed to through smoking^54^. Smoking bans in health centers can be considered punitive or discriminatory, further emphasizing the need to implement real psychological support programs^56^.

#### Factors supporting smoking cessation

Factors that can positively influence smoking cessation include the work environment in the pulmonary or oncology departments, where medical staff deal daily with smoking-related pathologies, as well as strong familial support, especially from spouses or children^58^. Pregnancy remains one of the most important motivators for quitting^58^. Other contributing factors include encouragement from colleagues or friends^54^, behavioral confidence^60^, exposure to chemical agents (as seen in laboratory settings), and engagement in regular physical activity^33^.

Passive smoking in the workplace among nurses is another important aspect to consider. A study by Xia et al.^61^ found a significant association between exposure to secondhand smoke, defined by exposure to cigarette smoke of more than 15 min per day per week for the past year, and declines in mental (OR=1.48; 95% CI: 1.07–2.07), physical (OR=1.51; 95% CI: 1.25–1.83), and overall health (OR=1.58; 95% CI: 1.3–1.93), based on the Cornell Medical Index (CMI)^61^. We also add here the findings of a study in Indonesia that identified passive smoking as a strong predictor of heart disease in employees with high blood pressure^62^.

#### Smoking cessation counseling practices among nurses

A study involving oncologists, family doctors and nurses identified that nurses, who may have more frequent and longer contact with patients, were more involved in identifying and advising smoking patients^28^.

Most nurses, about 90%, think they are models for patients. However, a study conducted in Turkey showed that about 30.7% of nurses include smokers, only about 57% ask the patient if they smoke, and 8.4% advise the patient to quit smoking, but the data varies by region^29^.

Although nurses have an advantageous position for advising patients on smoking cessation, the interventions they offer are frequently suboptimal. Most nurses, whether they are smokers or non-smokers, ask the patient about the smoking status. However, a statistically significant difference was identified in advising patients to quit smoking between smoking nurses and non-smokers, with smoking nurses being 13% less likely to advise their patients to quit^54,63^.

Because cardiovascular pathology is an important consequence of chronic smoking, a study was conducted among nurses in cardiology departments who identified that although all nurses consider quitting smoking to be a primary goal for patients, there are significant barriers both in their preparation for this purpose and in terms of insufficient time^64^. However, there is an opening of this category of staff in vocational training for the purpose of anti-smoking counseling. Additional training, developing self-confidence and implementing structural tools in medical practice are necessary goals to achieve among nurses^64^.

#### Educational and training interventions

Because the most well-known and important consequence of smoking is lung cancer, in China it was analyzed which is the best practice for screening, at that time there is uncertainty about the optimal criteria for implementing a program^65^. It has been shown that a method of making screening more effective is the involvement of nurses in counseling, pharmacological support and telephone follow-up of patients, which brings superior health benefits compared to isolated screening^65^. Thus, the study reported that a screening and counseling approach for people aged >45 years with a smoking history of more than 20 pack-years, including both active smokers and those who had quit smoking in the last 15 years, was associated with improved outcomes^65^.

Since emergency departments can provide an ideal educational window for anti-smoking interventions, it has been proposed to implement a program that is based on training nurses for a brief counseling of patients by ‘ask, advice and refer’. After being trained, nearly 76% of nurses felt fit to provide counseling^66^. During the study, 6.3% of patients identified as smokers accepted educational materials and referral to specialized smoking cessation programs, which strengthened the belief that this tactic is feasible, acceptable and effective^66^.

Another study evaluated the effectiveness of a personalized e-learning program that provided advice tailored to the level of knowledge, confidence and experience of each nurse, which led to significant improvement in compliance with smoking cessation counseling guidelines among experienced nurses^67^.

Another way of digital education was tested in the Czech Republic and Poland, and consisted of two 30 min webinars, the first about the role of nurses in smoking cessation, and the second about quitting smoking in an oncological context. Of the people involved, 60.83% were non-smokers, and 25.35% were former smokers^68^. They were found to have had a positive influence, with nurses being significantly more likely to intervene through anti-smoking counseling and recommending services specialized in smoking cessation. The percentage of nurses who had anti-smoking interventions in the last week increased significantly from 66% to 83%^68^. However, short educational interventions, although temporarily increase the knowledge and intention to advise patients for the purpose of smoking cessation, do not provide long-term behavior change, which emphasizes the need for periodic training^69^.

#### Smoking in psychiatric nursing settings

A study by Taniguchi et al.^70^ evaluated the effectiveness of a one-day educational program that included nurses from Japan, aiming to promote anti-smoking patient counseling. The program, which included presentations on the effects of smoking and nicotine addiction, practical exercises and role-playing games, led to a significant increase in smoking cessation counseling even 3 months after the intervention, although the study could be limited by a selection bias, with the likelihood of only responding to nurses who were already significantly more involved in patient well-being^70^.

A particularly important issue is that of smoking in psychiatric wards^71^. However, a Taiwanese study^71^ found low self-efficacy of psychiatric nurses in providing smoking cessation services, with an average score of 55.3/100. Approximately 80% of these nurses believed that smoking cessation worsens psychiatric symptoms such as depression, anxiety, insomnia, and irritability^72,73^. Many reported observing patients becoming calmer after smoking, and about a third admitted using cigarettes as a reward^72^.

Numerous barriers impede effective smoking cessation counseling. These include a lack of training in this aspect, insufficient time, passive exposure to smoking in the workplace, perceptions that smoking is used for self-medication^71^, insufficient social^74^ or institutional support^54^, resistance from patients, and the absence of clear organizational policies or adequate resources^74^. Additionally, some healthcare providers believe that patients are already aware of the risks and unlikely to change^75^ – especially among vulnerable groups (mental health problems, addictions, homeless people)^52^. However, the patient’s willingness to listen, empathic communication and family involvement can facilitate interventions on smoking patients. Additionally, it was found that higher cigarette taxes, media interventions and anti-smoking legislation applied can help to this end^75^.

### Smoking among medical students

An important population category to consider is that of students from the faculties of medicine. Effective strategies for quitting smoking, from this early stage, can lead to the formation of future professionals more aware of the risks of smoking and with a superior ability to effectively advise patients for smoking cessation^58^.

A notable proportion of medical students are smokers themselves, and study programs rarely address smoking as a personal medical problem. In Poland, a quarter of students are either smokers or former smokers, and three-quarters said their education did not cover the harmful consequences of smoking^76^.

The study by Chen et al.^77^ shows how smoking is widespread among medical students, especially in male students, with an increasing trend over the years of study. Almost 70% of male students and 27.6% of female students smoked at least at some point in life, of which 26.5% of men and 1.6% of women smoked in the last 30 days^77^. Identified potential risk factors included depression, low academic performance, and social influences, which highlight opportunities that can be addressed for preventive interventions, especially in the early years of college when the tobacco habit has not yet been strengthened^77^.

Another study of smoking among medical students conducted in Nepal highlights a worrying prevalence of smoking, with about a third of active smokers among third-year students, and almost half of the students who smoked at least once in their lives^78^. The same trend of high prevalence has been identified this time, with a much higher frequency in men than in women. A percentage that reaches close to 24% of them initiated this behavior aged between 20–24 years, which indicates that smoking initiation occurred during the period corresponding to medical training for a substantial proportion of participants^78^. Unfortunately, only 28.3% received formal training for smoking cessation. An encouraging percentage of them (96%), regardless of their smoking status, considered the role of medical staff in the smoking cessation of patients to be important^78^.

Vinnikov et al.^79^ tracked the prevalence of smoking among medical students, identifying the highest prevalence of smokers in year 6, nearly 86% among male students, and 58% among women, which shows, contrary to expectations, that several years of study were associated with reduced confidence in the causal relationship between smoking and chronic disease. Only 69% of students recognized the clear link between smoking and associated comorbidities. The overall prevalence among all medical students was 35%, but after biochemical validation by measuring expired CO (carbon monoxide), the actual prevalence of smoking reached almost 45%^79^. Although the level of nicotine dependence proved to be low according to the Fagerström test, the intention of smoking cessation was weak, only about 23% being willing to quit^79^. A high smoking prevalence was also reported among students in Italy, with Pianori et al.^53^ identifying 45.45% of the 22 students included in the study as current smokers^53^.

Ferrante et al.^80^ looked at the general attitudes of medical students about the role of medical professionals, identifying that they generally agreed that they should receive specific training for quitting smoking, although during the faculty only 21.3% received; 96% agreed that professionals should recommend smoking cessation to patients, and almost 68% agreed that medical staff should be a behavioral model for patients^80^. Smoking has also been shown to be more common among students in nursing, physiotherapy and pharmacy (49.2%) than in general or dental medicine (29.5%)^80^.

### Smoking among pharmacists

Pharmacists represent another key group of healthcare professionals who can significantly contribute to patient education. Steed et al.^81^ proposed a strategy to support smoking cessation in pharmacies, ideal spaces for health promotion, based on two 150 min training sessions focused on patient-centered communication skills and behavior change techniques. Although there were barriers such as lack of time and financial motivation for pharmacies, stereotypes about who has the ability to quit, lack of involvement of all staff, lack of a private place for counseling, as well as poor course programming, both as location and as hours, intervention was perceived as acceptable and useful, while increasing self-efficacy of staff after training^81^.

Another study in the United States assessed the integration of anti-smoking education into pharmaceutical doctoral programs, with a view to expanding the role of the pharmacist in prescribing smoking cessation drugs^82^. Almost all faculties include thematic content in courses, the most commonly addressed topics being smoking cessation pharmacotherapy, anti-smoking counseling and nicotine pharmacology through courses that include, among others, case studies, role-play activities, Power Point slides or instructional videos^82^.

### Findings

This narrative review highlights the variability in smoking prevalence among healthcare professionals, depending on geographical location and professional category. Higher rates have been observed in some groups, particularly among nurses and auxiliary staff^20,22,63^. The spread of smoking-related habits has been associated with sociodemographic factors such as gender, age and education level^20,23,76^. Considerable gaps in smoking cessation counseling practices have also been identified, despite the professional responsibility of healthcare professionals in tobacco control^28,30,45,63^. Considerable heterogeneity was also observed across studies in terms of design and reported prevalence.

These findings may be explained by organizational and cultural factors, workplace anti-smoking policies, and the availability of resources for smoking cessation counseling. The lower prevalence among doctors compared with other professions may reflect the importance of medical education in their own smoking behavior^20,22^. Professional stress, which includes shift work or emotional strain, but also significant events (example: COVID-19 pandemic) are frequently reported as triggers to initiate or maintain smoking, supporting the need for interventions focused on non-nicotine-based coping strategies^24-26^.

Smoking is not consistently addressed in clinical practice^29^. Although many clinicians ask patients about their smoking status, active advice and prescribing pharmacotherapy remain suboptimal and documentation of smoking status remains inconsistent^29^. Lack of time, compensation and standardized materials are a hindrance to patient counseling^29,36^.

Curricular gaps reduce the trust and quality of counseling. Interactive training sessions (role-play, personalized e-learning) seem more effective than passive didactics, but require periodic repetition to support behavior change^67,82^. Dedicated workplace programs can produce meaningful quit rates and organizational benefits (low absenteeism)^43^. Integration of the ‘ask-advise-refer’ model in emergency settings and involvement of nurses in screening/tracking, increase coverage of interventions. In the future, multicenter longitudinal studies, cost-effectiveness assessments of institutional anti-smoking programs, and digitally supported interventions (e.g. telephone follow-up) for healthcare professionals can be considered.

### Limitations

Despite offering an updated synthesis of available evidence, the present review is subject to several limitations. First, given that it is a narrative review, the selection and synthesis of the studies were not based on a systematic protocol, which leads to an increased risk of selection bias. Secondly, there was substantial heterogeneity among the included studies, notably through the design of studies, sample characteristics, geographical distribution and interpretation of smoking-related behavior, leading to limited comparability across contexts. Many studies have been based on self-reported questionnaires, which may have resulted in reporting bias and social desirability bias, as healthcare professionals could have under-reported smoking-related behaviors. Furthermore, the screening and selection of studies were performed by a single reviewer, and the methodological quality of the included studies was not formally evaluated.

In addition, a possible publication bias cannot be excluded, given that studies reporting higher prevalence or significant results are more likely to be published. Finally, causal factors cannot be established between the identified determinants and the smoking behavior among healthcare professionals due to the predominantly cross-sectional design of the selected studies. Although most of the included articles were published in English, two studies in French were also included. Nevertheless, relevant studies published in other languages may not have been identified.

## Conclusion

Tobacco use among healthcare professionals remains a major problem despite the high level of knowledge of its risks. In addition to the impact on their own health, smoking among medical staff can affect their credibility in the eyes of patients and reduce the effectiveness of smoking cessation counseling.

Determinants of smoking, including professional stress, insufficient training and inadequate organization of counseling services, highlight the complexity of smoking behavior in medical personnel.

Thus, additional multicenter studies with a well-designed methodology are needed to gain a more comprehensive understanding of the determinants of smoking behavior among health professionals and to assess the effectiveness of prevention strategies. Strengthening the evidence base in this area is essential to provide a solid foundation for future public health policies and appropriate vocational training.
